# Supplementary material for: Are carbon emissions trading and green financial instruments synergistic? -Comprehensive quantitative research based on content analysis
Source: PLoS One. 2024 Mar 7;19(3):e0298601. doi: 10.1371/journal.pone.0298601 (PMC10919601; doi:10.1371/journal.pone.0298601)
Supplement: S1 Data — (ZIP) [file pone.0298601.s001.zip › Data/地级市Z_F工作报告.zip!/╣π╢1⁄2/╔╟═╖╩╨/└┤╫╘╣1⁄2╓┌║┼í╛┬φ┐╦╩2╛▌═°í┐_2007.docx]

各位代表：

　　我代表汕头市人民政府向大会作政府工作报告，请予审议，并请政协各位委员和其他列席人员提出意见。

　 工作回顾

　本届政府2003年6月任职以来，在省委、省政府和市委的正确领导下，坚持以邓小平理论和“三个代表”重要思想为指导，全面落实科学发展观，励精图治，真抓实干，着力解决发展中的主要问题和主要矛盾，经济社会发展加快，精神文明和民主法制建设取得新成效，出现了稳定发展的新局面。

　一、经济保持持续发展

　　2006年全市生产总值741亿元，比2005年增长11.6%，2003年至2006年年均增长10.7%；地方财政一般预算收入34.93亿元，比增18.6%，年均增长14.9%。

　工业经济实力增强。2006年全市工业总产值1469.9亿元，比2002年增长60.8%。规模以上工业企业1535家，比2002年增加642家，产值占工业总产值的比重提高了17个百分点。能源、石化等战略性产业引进力度加大，装备制造、纺织服装等八大支柱产业发展壮大。创建机械装备、包装印刷生产开发等国家级产业基地。整合工业园区，壮大产业集群。建筑业稳步发展，完成施工产值466亿元。

　效益农业势头良好。2006年全市农业总产值84.24亿元，比2002年增长4.6%。规划建设五大优势农业区，加快推进农业产业化，科技兴农效益明显，粮食专业化规模化生产有新突破。

　服务业明显提升。2006年第三产业增加值309.5亿元，比2002年增长44.1%。港口货物吞吐量2012.5万吨，集装箱吞吐量44.2万标箱，创历史新高。消费活跃，市场繁荣，社会消费品零售总额年均增长13.6%。信息服务业、房地产业加快发展。

　区县经济协调发展。金平、龙湖、澄海区保持较快增速，濠江、潮阳、潮南区发展加快，南澳县生态旅游经济呈现良好态势。特色经济成为区县经济的支撑力量，全市有省级专业镇22个，居全省地级市第二位。

　二、城市规划建设迈出新步伐

　城市规划全面展开。开展城市和产业发展战略定位研究，完成城市发展概念性规划、城市近期建设规划、海洋功能区划、广澳港区总体规划等一批涉及全局的重大规划，完成干线公路网规划、商业网点规划、绿地系统规划等一批专项规划。

　三大经济带规划稳步推进。规划东部城市经济带、工业经济带、生态经济带，打造城市发展新格局。东部城市经济带已完成河口整治项目可行性研究报告，正抓紧申报立项。工业经济带完成控制与发展规划，着手规划一批专业园区。生态经济带进入调研论证阶段。

　基础设施建设进展加快。建成华能汕头电厂二期工程等一批能源基础设施，珠池港区二期工程、汕汾高速公路、金鸿公路等一批交通基础设施，牛田洋海堤、秋风岭水库等一批水利基础设施，雷打石生活垃圾填埋场二期、部分市政道路连接段和“水浸街”改造等一批市政工程。金凤路桥、金砂路东延路段、广澳港区一期工程、中山公园改造、龙湖沟整治等项目继续推进。

　城市环境得到改善。巩固和发展国家卫生城市、优秀旅游城市工作成果，加强国家环境保护模范城市复查整改工作，积极创建国家园林城市，城乡环境质量良好。城市建成区人均公共绿地面积11平方米。林业生态建设加快，海岸湿地自然保护区成为国内首个国际湿地示范区。

　三、经济发展活力进一步增强

　自主创新能力提高。2006年全市高新技术产品产值229亿元，比2002年增长90.8%。新增省级以上高新技术企业88家，省级工程研发中心12家。被确定为“国家知识产权试点城市”。新增中国名牌产品8个，中国驰名商标7件，国家免检产品26个，国家出口免验产品1个，国家级区域性品牌7个，荣获“中国品牌经济城市”称号。

　各项改革不断深化。建立国有资产监督管理新体制，完成60家市直国有企业产权改革。调整文化广电新闻出版管理体制，文化体制改革综合试点工作步伐加快。推进投融资体制改革，一批城市基础设施项目采用市场化模式运营。推进农村信用社改革试点工作。供电、供水、医疗价格改革和路桥收费年票制改革收效明显。

　内外源型经济持续发展。投资促进机制不断完善，被评为“中国投资环境百佳城市”。加大招商引资力度，实际吸收外商直接投资3.76亿美元。积极扩大出口，2006年外贸出口34.8亿美元，比2002年增长122%。民营经济加快发展，民营投资占全社会投资比重连续三年超过40%，民营工业企业产值占全市工业总产值的63%。

　四、经济社会管理不断改善

　财税管理进一步加强。全面开展经济普查，规范企业统计管理。推进财政支出管理改革，全市1072个单位实行部门预算，580个单位纳入国库集中支付管理。全面清理行政事业单位资金资产，加强国债和财政投资重点项目财务监管，确保国有资产资金安全。强化税收征管，税收收入占生产总值比重逐年提高。

资源管理和利用初见成效。加强土地管理，盘活存量土地1万亩。加强水资源保护和江河整治。大力节能降耗。发展循环经济，潮阳区贵屿镇被列为全国循环经济试点单位，南澳清洁能源产业较快发展。被列为省建设节约型社会试点城市。

　经济社会秩序逐步规范。打击各种经济违法犯罪行为，加强商业贿赂治理，市场经济秩序进一步规范。严厉打击暴力性犯罪、“两抢一盗”和“黄赌毒”违法犯罪行为。推进治安防控体系建设，治安长效机制逐步形成。落实防范重大安全事故责任制，开展火灾隐患排查整改，加大安全生产投入，安全生产形势有所好转。

　一批社会矛盾得到有效化解。认真执行《信访条例》，加强信访工作。建立健全社会稳定预警机制、矛盾纠纷排查调处机制、突发公共事件应急管理机制。妥善处理一批历史遗留问题。继续寻求汕头市商业银行处置办法。积极解决历史拖欠征地补偿款、建设领域政府项目拖欠工程款等问题。

　五、和谐社会建设扎实推进

　人民生活水平有所提高。2006年中心城区居民人均可支配收入10949元，农村居民人均纯收入4450元，分别比2002年增长25.3%和14.3%。城乡居民储蓄存款余额850亿元，比2002年增长80.7%。物价稳定，居民消费价格指数控制在合理上涨区间。中心城区人均住房面积27.06平方米，比2002年增加7.74平方米。全市电话用户超过480万。

　一批热点难点问题得到解决。抵御台风“珍珠”等自然灾害袭击，救灾复产工作成效显著。深化农村税费改革，全面免征农业税，农民负担减轻99.15%。解决70万人“饮水难”问题。完成141个行政村217公里水泥路建设。提供廉租公房2046套，完成农村贫困户危房改造2509户，修建农村低保户住房400套。解决商品房确权发证遗留问题，产权登记发证2.5万套。建成星光老年之家162个。为残疾人办实事，荣获“全国残疾人工作示范城市”称号。

　就业和社会保障工作扎实开展。实施积极就业政策，2006年城镇登记失业率3.1%。扩大社会保险覆盖面，实现社会保险市级统筹，养老保险基金当期收支平衡。社会福利事业加快发展。城乡社会救助体系建设稳步推进，累计有23万人享受最低生活保障，基本实现应保尽保。

　各项事业全面进步。新建改建扩建中小学校舍127万平方米，增加学位18.9万个。汕头一中新校区建成投入使用。实施农村免费义务教育，受益学生74.5万人。职业教育、成人教育和民办教育稳步发展，汕头大学和汕头职业技术学院办学水平不断提高。文化大市建设步伐加快，潮剧发展和改革初见成效，市图书馆、博物馆新馆投入使用。城乡公共卫生体系逐步完善，市疾病预防控制中心主体工程完工，非典、人禽流感等重大传染病得到有效防控。新型农村合作医疗参合率37.4%。体育事业蓬勃发展，实现奥运会金牌零的突破。人口和计划生育工作有新进步。殡改火化率连续四年达标。民族、宗教、人防、外事、侨务、对台、地震、档案、保密、方志等工作取得新成绩。

　精神文明建设巩固发展。群众性精神文明创建活动稳步推进，现代公民教育初显成效。青年学生和未成年人思想道德建设得到加强，关心下一代工作力度加大。普法宣传和法律服务工作扎实开展，全民法律意识普遍提高。慈善事业进一步发展，扶贫济困蔚然成风。国防教育、国防后备力量建设和双拥优抚安置工作继续加强，蝉联“全国双拥模范城”称号。

　六、政府自身建设继续加强

　民主法制建设取得新成果。认真执行市人大及其常委会各项决议并向其报告工作，坚持向人民政协通报情况，自觉接受监督。办理人大代表议案、建议315件，政协提案683件。认真行使立法权和政府规章制定权，提请市人大常委会审议法规草案10件，颁布政府规章14件。加强与工会、共青团、妇联等群众团体的联系，广泛听取社会各界的意见和建议。全面完成村（居）委会换届选举。

　依法行政工作取得新成绩。修订实施《汕头市人民政府工作规则》，坚持重大问题集体研究，涉及人民群众利益的重大决策实行听证制度和公示制度。实施重大行政决策和规范性文件事前法律审查制度，推进政府决策法制化。全面清理行政执法主体、执法依据和执法行为，规范行政执法。加强审计监督、行政监察和政府层级监督，推进行政复议工作。

　行政管理体制改革取得新进展。完成第三轮行政审批制度改革，削减审批事项161项。建立健全政府投资项目顾问、公示、决策、重大投资项目稽查和责任追究等制度，严格执行建设工程招投标、土地招标拍卖挂牌出让、产权交易、政府采购等制度。

　政风建设取得新成效。开展改进机关作风、提高行政效能、优化投资环境等专项活动，行政效率得到提高。落实廉政建设责任制，严肃查处违法违纪案件，认真纠正损害群众利益的不正之风，切实加强领导干部离任经济责任审计。县级以上政权机关政务公开全面推行，全市电子政务网络平台初步建成，市政府新闻发布制度逐步完善。

　各位代表！我市经济社会发展取得的成绩，是省委、省政府和市委正确领导的结果，是各级人大及其常委会和人民政协监督支持的结果，是全市人民同心同德、奋力拼搏的结果，也是社会各方面支持帮助的结果。在此，我代表市人民政府，向全市广大工人、农民、知识分子、干部职工、驻汕人民解放军、武警官兵、人民警察和社会各界表示崇高的敬意！向关心和支持汕头建设的港澳台同胞、海外侨胞和国际友人表示衷心的感谢！

　我们也清醒地认识到，发展中还存在许多突出的矛盾和问题：一是经济增长速度偏慢、增长方式粗放、增长动力不足，产业层次较低，加快发展的任务很重；二是经济发展与人口、资源、环境的矛盾突出，土地等制约因素日益凸显，可持续发展的任务很重；三是城乡二元结构明显，协调发展的任务很重；四是社会治安不容乐观，安全生产形势严峻，城市管理亟待加强，一些重大历史遗留问题尚未解决，经济社会管理的任务很重；五是影响发展的体制机制问题依然突出，行政效率还不高，优化投资软环境的任务很重；六是财政收支矛盾突出，公共设施投入不足，改善公共服务的任务很重，等等。对这些问题，我们将高度重视，努力解决。

　各位代表！回顾三年多来的实践，我们深深体会到：

　　———必须坚持发展第一要务，促进加快发展。要以科学发展观统领全局，强化能快则快的发展意识，加快经济结构调整，切实转变经济增长方式，推动经济又好又快发展。

　　———必须坚持环境保护和资源节约，促进可持续发展。要立足当前，着眼长远，注重经济发展与人口、资源、环境相协调，节约集约利用资源，发展循环经济，保护生态环境，努力建设资源节约型、环境友好型社会，促进经济社会可持续发展。

　　———必须坚持加强公共管理，促进有序发展。要依法加强经济和社会管理，规范市场经济秩序，强化社会治安和安全生产管理，营造文明法治环境，以管理促发展。

　　———必须坚持稳定压倒一切，促进稳定发展。要正确处理好改革、发展、稳定的关系，保持高度的政治敏感性，努力提高危机处理和应急管理能力，妥善处理历史遗留问题，积极化解各种矛盾和不安定因素，确保经济社会稳定发展。

　　———必须坚持理顺内外关系，促进合力发展。要着力加快体制机制创新，优化投资软环境，发挥侨乡优势和民资优势，调动一切积极因素，汇聚一切资源，团结一切力量，形成发展的强大合力。

　　———必须坚持以人为本，促进和谐发展。要努力维护好、实现好、发展好最广大人民群众的根本利益，真心实意为民办实事好事，千方百计解决关系群众切身利益的现实问题，让广大人民群众共享改革和发展的成果。

　 今后五年的奋斗目标和工作安排

　今后五年，是我市全面落实科学发展观、推进和谐社会建设的重要时期，更是我市寻求新突破、谋求大发展的关键时期。

　进入新的历史时期，我们迎来了难得的发展机遇。省委、省政府促进粤东地区加快经济社会发展工作会议的召开，给汕头的发展带来了强大的动力。经过近年来的艰苦努力，我市经济呈现加快发展的趋势，经济社会管理日趋规范，社会更加和谐稳定。汕头作为经济特区、著名侨乡、沿海开放城市、粤东中心城市，具有独特的区位、优良的港口、日臻完善的基础设施、丰富的民资民力，发展的潜力和空间很大。我们的干部队伍素质比较好，全市上下谋求发展的愿望十分强烈。我们要看到优势，看到希望，抢抓机遇，加快发展。我们相信，只要全市人民树立信心，振奋精神，同心同德，奋力拼搏，汕头就一定能发展得更好！

　今后五年政府工作的基本思路是：以邓小平理论和“三个代表”重要思想为指导，全面落实科学发展观，全面构建和谐社会，按照“三年打基础、五年大变化、十年大发展”的总体部署，围绕建设现代化港口城市、区域性中心城市、生态型海滨城市的目标，以加快发展作为全部工作的主题，全力实施可持续发展、工业强市、以港兴市、开放带动、自主创新五大发展战略，着力提高自主创新能力，着力调整优化经济结构，着力转变经济增长方式，着力加强经济社会管理，着力改善人民生活，不断推进经济建设、政治建设、文化建设和社会建设，把经济社会发展切实转入科学发展轨道，努力实现经济社会又好又快发展。

　经过五年的努力，到2011年，全市经济实力明显增强，生产总值达到1400亿元，年均增长14%以上，增速达到全省平均水平，单位生产总值综合能耗比“十五”期末降低16%以上，一般预算收入占生产总值比重提高到6%以上；城市功能明显提升，东部城市经济带建设全面启动，工业经济带稳步推进，重点基础设施建设全面铺开，城市空间架构拉大，城市功能布局更加科学合理，全市城镇建成区面积达335平方公里；人民生活质量明显提高，城镇居民人均可支配收入和农民人均纯收入分别年均增长6%和4%左右，人居环境有较大改善，社会就业比较充分，基本建立起覆盖城乡、比较完善的社会保障体系；和谐社会建设取得明显成效，社会各项事业全面发展，社会管理更加有序，城乡文明程度明显提高。2007年，是新一届政府的届首之年。做好今年的各项工作，对于实现今后五年工作目标至关重要。综合考虑诸多因素，今年全市经济和社会发展的主要预期目标是：生产总值增长12.5%，工业总产值增长14.5%，单位生产总值综合能耗下降3.2%，社会消费品零售总额增长15%，全社会固定资产投资额增长16%，外贸出口总额增长12%，居民消费价格指数涨幅控制在3%左右，地方财政一般预算收入增长14.5%，城镇登记失业率控制在3.5%以内，常住人口自然增长率控制在7.2‰以下，城镇居民人均可支配收入增长6%，农村居民人均纯收入增长5%。

　为实现上述目标，要做好九方面工作：

　一、加快城市规划建设，高起点打造城市发展新格局

　完善城市规划编制。修编城市总体规划、土地利用总体规划，整合市域各区总体规划，严格按照总体规划管理和实施。编制重点区域控制性详细规划，加紧编制东湖教育园区、梅溪河—乌桥岛沿岸控制性详细规划。加快南滨—葛洲片区开发，抓紧北山湾片区规划调整，着手开展招商等工作，推进南区建设。完成汕头港总体规划修编。

　高标准规划建设三大经济带。东部城市经济带重点布局金融、贸易、商务、文化及房地产业，逐步建设成为新的城市中心。今年抓紧河口整治工程项目报批，启动新津河口片区、澄海塔岗围片区建设。工业经济带争取引进大项目，推动台商投资区、珠三角产业转移园区、珠宝工业城等专业园区的实施，重点布局石化、能源、装备制造等临港工业。生态经济带抓紧规划编制，以点带面，分步实施，重点布局生态农业、水产养殖、旅游观光、高新技术、教育培训等生态型产业。

　抓紧基础设施建设。重点加快广澳港区建设，完成2个5万吨级码头建设，争取5万吨级航道和5万吨级石油化工码头尽快开工，启动10万吨级集装箱码头、30万吨级原油码头、粤东煤炭中转码头、西防波堤工程前期工作。完善澄海、潮阳、潮南、南澳港区规划，启动潮阳港区建设。配合做好厦深铁路建设，加快规划广澳港区疏港铁路。着手做好过海隧道、第三跨海大桥的比选论证工作。加快建设汕头至揭阳、汕头至普宁高速公路，开展惠揭高速公路、登洪高速公路汕头段前期工作。加快国省道改造和客货运枢纽站场建设，加紧建设南澳大桥，建设陈沙公路。加快金凤路桥、中山东路、大学路、机场路等城市主要出入口道路建设，改造海滨路、中山西路、金砂路和迴澜桥，贯通一批市政道路连接段，续建汕樟北路，建设濠州路。加大旧城和城中村改造力度，推进小公园改造项目建设。加快城市管道燃气建设。加快成田、谷饶、金凤输变电工程和华能电厂至月浦站线路工程建设，争取500千伏潮南输变电工程尽快开工，今年开工建设南澳110千伏过海电缆工程。加快推进汕头大围等江海堤围达标加固工程、莲阳桥闸重建工程以及潮阳、潮南、南澳引韩供水工程。

　二、走新型工业化道路，推动产业结构优化升级

　提高自主创新能力。整合全社会科技资源，鼓励发展风险投资，完善科技和产业服务支撑体系。实施品牌带动战略，鼓励企业争创名牌产品，进一步扩大名牌效应。加大拥有自主知识产权的核心技术和关键技术研发力度，培育发展创新型企业。着力推进“国家知识产权试点城市”、澄海“创建区域国际品牌试点地区”建设。加快构建公共科技创新平台体系，推进特色产业基地建设。加快技术创新专业镇建设。

　强化工业主导地位。加强工业的组织领导，明确优先发展产业，加强产业投资引导，用好省产业引导资金。完善现有工业园区，提高园区承载能力，推进产业集群升级示范区建设，壮大特色产业集群。重点发展光机电一体化、电子信息、机械装备、生物工程、新材料等产业。加大技术改造力度，推动传统产业优化升级。发挥高新区政策优势，扩大发展规模，引导高新技术产业集聚。做强做大工业企业，争取到2011年，规模以上工业产值占全市工业总产值的比重达到80%左右。

　发展临港产业。实施以港兴市战略，优化港口结构，充分利用岸线资源和港口资源，重点发展石化、能源等战略性产业。推动昊华化工集团重组海洋集团，全力支持韩国SK集团聚苯乙烯树脂项目尽快投产。加快华能海门电厂1号、2号机组建设，争取3号、4号机组“十一五”期末开工建设。争取海门60万千瓦燃煤电厂尽快立项建设。发挥保税区政策优势，深化功能开发，实施区港联动，大力发展临港工业和港口物流业。

　加快发展综合服务业。建设特色商业街，推行连锁经营、仓储超市、专业配送等组织形式和服务方式，培育和发展大型商贸企业。抓紧规划建设美食城、珠宝商城、南滨会展中心，规划建设工艺玩具、纺织服装等大型专业市场。积极发展通讯、软件等新型服务业。发展资本市场，拓展金融保险和证券业。整合旅游资源，培育发展特色滨海休闲、度假、商务旅游，建设区域性旅游中心城市。发挥“建筑之乡”优势，支持建筑业开拓国内外市场。积极发展经济适用房、廉租公房和普通商品住宅，促进房地产市场健康发展。

　三、发展农村经济，推进社会主义新农村建设

　发展现代效益农业。全面推进五大优势农业区建设，积极发展蔬菜、禽畜、水果、水产等特色优势农产品，壮大主导产品和支柱产业。培育发展花卉园艺业、观光农业、生态农业和休闲渔业，加快发展加工农业和流通农业。大力发展海水养殖业、外海捕捞业。壮大农业龙头企业，培育农民专业合作经济组织，提高农业产业化水平。依托优势农业产业带和特色种养基地，大力发展农产品精深加工和专用粮油食品加工。强化农业动植物疫情防控工作，加快农业科技体系和农产品质量安全体系建设，办好农业现代化示范园区。加强对台农业科技交流与合作，积极争取创办台湾农民创业园和台湾农产品集散中心。

　壮大镇域经济。以民营经济为基础、以工业化为核心，发挥中心镇的辐射带动作用，引导各镇培育具有地方特色、市场竞争力强的优势产业，提高工业化、城镇化水平。提升农村商贸、餐饮、运输等传统服务业水平，加快推进“万村千乡市场工程”、信息咨询、社区中介服务、金融保险等现代服务业。

　改善农村基础条件。加强农田基础设施和山塘水库除险加固工程建设，提高农业抵御自然灾害能力和综合生产能力。加快海门国家中心渔港建设，抓紧申报云澳国家中心渔港、达濠国家一级渔港建设项目，建设现代渔港经济区。完善农村电网、有线电视、通讯和信息网络设施建设，加快农村信息化。加强农村道路及公共交通配套设施建设。积极开展创建生态文明村活动，改善农村生态环境。推进以“改路、改水、改房、改厕、改灶”和村庄绿化为主要内容的农村环境整治。稳步推进农村社区建设。开展创建文明村镇、文明家庭活动。

　四、深化改革开放，增强经济发展动力

　进一步深化各项改革。以产权制度改革为核心，以强化财务管理为突破口，以职工安置为重点，推进国有企业改革。对长期亏损、资不抵债、不能清偿到期债务的国有企业，实施依法债务重组、资产托管、破产关闭、解散或停业整顿等措施，加快产权制度改革步伐。鼓励民营企业、外资企业参与国有企业改革。大力培育、扶持优质企业改制上市。加强政府债务和资产管理，规范政府举债行为。积极稳妥推进汕头市商业银行等地方中小金融机构处置工作，继续推进农村信用社改革试点工作。推进资源和公用事业价格改革。

　壮大民营经济。实行同等待遇，放宽市场准入，鼓励和支持民营资本进入基础设施、公用事业及法律法规未禁止的其他行业和领域，把扶持民营经济发展的政策措施落到实处。支持民营企业体制创新、科技创新和管理创新，不断提高民营企业素质，提升民营经济产业层次。构建面向中小企业的信用担保体系和服务体系，积极帮助企业解决融资、用地、用工等困难和问题。培育和促进行业协会发展，引导民营企业自主发展和自律管理。

　优化提升外源型经济。精心策划各类大型招商引资经贸活动，突出抓好重点产业招商，争取引进一批项目。积极争取在外潮商回乡投资创业。鼓励企业增资扩产。鼓励和扶持拥有自主知识产权、自主品牌的商品出口，扩大传统优势产品出口，增加高附加值产品出口。巩固传统出口市场，大力开发俄罗斯、非洲、中东、拉美等新兴市场。调整和优化外贸出口经营主体结构，鼓励中小企业直接参与国际竞争。积极应对各类国际贸易摩擦。优化进口产品结构，扩大进口。发挥海关、检验检疫局等口岸单位作用，加快大通关建设。

　加强区域经济合作。加强与粤东各市在产业布局、基础设施建设、资源开发等方面的协作。积极参与泛珠三角、港澳台地区、中国—东盟自由贸易区等的经济合作。加强与闽粤赣经济协作区、海峡西岸经济区在科技、信息、文化、旅游、人才等方面的交流合作。

　五、提高服务和管理水平，不断优化投资软环境

　加强服务环境建设。转变服务观念，创新服务方式，改进工作作风，提高办事效率，更好地为基层、企业和社会公众服务。在市企业投资管理服务中心的基础上，按照“一楼式”集中审批和“一站式”电子政务相结合的运作模式，建立健全体制机制，加强行政效能监察，推进行政服务中心建设。

　加强市场环境建设。建立和完善与信用相关的政策法规，健全社会信用体系。改善金融服务，增加信贷支持，防范化解金融风险。加强税收征管，促进税收增长与经济增长相协调。加强民间组织管理。加大市场监管力度，强化食品药品安全、知识产权保护等工作，严厉打击扰乱市场经济秩序的违法犯罪行为。

　加强市容环境建设。拓宽投融资渠道，加快城市公用设施建设步伐。改造城区排水系统，减少内涝现象。推进园林基础设施和城市路灯、步道、地下管网、环卫设施改造建设。落实管理责任制，加大执法力度，大力整治脏乱差，提高城市管理水平。开展城市管理行政执法进社区试点工作。整合城市公共资源，规范户外广告、停车场、咪表停车管理。今年继续推进龙湖沟整治及中山公园改造等工程。

　加强治安环境建设。开展平安汕头建设，强化社会治安综合治理，加强社会治安防控体系建设。集中整治治安重点地区和突出问题，加强出租屋和流动人口的管理，重拳打击“两抢一盗”等多发性犯罪，深入开展打黑除恶专项斗争，增强人民群众的安全感。严厉打击“黄赌毒”、“六合彩”等违法犯罪活动。高度重视信访工作，进一步完善社会矛盾纠纷排查调处机制、群体性事件预警处置机制，妥善处置群体性、突发性事件。

　加强人文环境建设。发扬吃苦耐劳、自强不息、坚韧不拔的优良传统，弘扬敢闯敢干、敢为人先、开拓进取的创业精神。深入开展社会主义荣辱观教育，抓好青年学生和未成年人思想道德建设，提高全民道德素质。深入开展群众性和谐创建活动，营造相互关爱、谅解宽容、服务社会的氛围。

　六、建设节约型社会，促进人口资源环境协调发展

　建设节约型社会。加强土地规划管理与控制，严格保护耕地。积极推进集体建设用地流转，整合盘活存量土地，节约集约利用土地。提高水资源管理和利用水平。广泛开展节能降耗活动，加强重点耗能企业的监督管理。

　加强环境保护和生态建设。制定实施全市环境保护规划，严格建设项目环保准入制度，实行排污许可制度，加强环保执法监督。巩固中心城区国家卫生城市、环保模范城市、优秀旅游城市创建成果，积极创建国家园林城市，加快澄海、潮阳、潮南三个新区建成区创建步伐。推进治污保洁工程建设，提高污水和垃圾无害化处理率。积极开展生态示范区创建活动，保护汕头湾生态环境，保护性开发海域、岸线，加强风景名胜资源保护。保护森林资源，加强水源涵养林、沿海防护林建设。保护韩江水质，整治榕江、练江等重点流域环境污染。切实推进殡葬改革工作。

　积极发展循环经济。开发建立“绿色技术支撑体系”，推广节能增效、资源综合利用先进技术。加强组织引导，推进贵屿国家循环经济试点建设。发展壮大南澳风电产业，建设清洁能源生产基地。

　加强人口和计划生育管理。突出抓好纯二女户结扎和计划生育优质服务，兑现奖励政策，严肃查处失职行为，稳定低生育水平，提高出生人口素质。综合治理出生人口性别比偏高问题，切实维护人口安全。

　七、突出发展教育卫生文化事业，促进社会全面进步

　大力发展教育人才事业。优先保障教育特别是义务教育的投入，落实农村义务教育经费保障机制。调整中小学布局，推进义务教育规范化学校建设，进一步提高“普九”水平。加快发展高中阶段教育，增加高中优质学位，力争2010年高中阶段毛入学率达到80%。大力发展职业技术教育和高等教育，启动滨职业技术学校新校区建设，提高汕头职业技术学院办学质量和效益。支持办好汕头大学和粤东高级技工学校，进一步办好汕头电大业大职大。积极发展学前教育、特殊教育、继续教育，规范发展民办教育。加强师资队伍建设，提高师资特别是农村师资水平。重视学校安全管理，保障师生权益。加强人才资源建设，创新人才工作机制，建立和完善人才市场机制，积极吸引优秀人才，营造优秀人才脱颖而出、充分发挥作用的社会环境。

　建设覆盖城乡居民的基本卫生保障制度。推进医药价格改革，加快完善有利于群众及时就医、安全用药、合理负担的医疗卫生制度体系。合理调整中心城区医疗卫生机构布局，大力发展城市社区卫生服务。巩固完善农村三级医疗预防保健网络，深化乡镇卫生管理体制改革，强化乡镇卫生院公益性职能。加强农村规范化卫生站建设，为群众提供基本医疗卫生服务。完善新型农村合作医疗制度，今年实现全市新型农村合作医疗参合率80%以上。加强公共卫生体系建设，提高重大疾病预防控制能力和医疗救治能力。大力推进中医药强市建设。

　发展文化体育等各项事业。弘扬优秀传统文化，继续推进潮剧改革与发展，加强文化遗产保护。加强文化基础设施建设，构建覆盖城乡的公共文化服务体系。发展广播电视和新闻出版事业。加快发展文化产业。推进体育事业和体育产业发展，办好第十一届市运会。加强现代国防教育，扎实开展双拥工作。加快发展妇女儿童事业，切实保障妇女儿童合法权益。重视和做好民族、宗教、统计、人防、外事、侨务、气象、地震、档案、保密、方志等工作。

　八、坚持为民务实，维护社会和谐稳定

　努力扩大就业再就业工作。完善和落实扶持再就业优惠政策，做好城镇就业和进城农民工就业工作，扩大与促进就业。到2011年底，力争全市就业规模达到264万人。加快建立城乡统一的公共就业服务组织体系，完善统一规范开放的人力资源市场。建立健全困难群众就业援助制度。实施“智力扶贫工程”、“十万农村青年技能培训工程”和退役士兵免费职业技能培训工程。维护劳动者合法权益，构建和谐劳动关系。

　完善社会保障体系。强化社保费征缴和社保基金管理，提升基金保障水平和抗风险能力，确保社保资金安全稳定运行。改革完善养老金计发办法，逐步做实养老保险个人账户。以非公有制企业从业人员为重点，扩大社会保险覆盖面。探索建立以失地农民为重点的社会保险制度。全面落实城乡居民最低生活保障制度，建立健全社会救助体系，实现应保尽保。积极发展残疾人事业、老龄事业、福利事业、慈善事业。

　解决群众关注的热点难点问题。大力发展城市公共交通，整治交通秩序，缓解城市交通拥堵状况。积极实施农村安居工程，加快农村贫困户危房改造。解决农村饮水安全问题。加大扶贫开发力度。加强城镇房屋拆迁管理和规划建设。加强农村征地管理，推行村务公开，维护农村社会稳定。健全安全生产体制机制和政策措施，加大投入，深化重点领域专项整治与监管，坚决遏制重特大安全事故，切实预防和减少人员伤亡和财产损失。

　九、加强自身建设，打造人民满意政府

　依法行政，建设法治政府。健全重大决策的规则和程序，实行依法决策、科学决策和民主决策。严格依法行使权力，善于依法管理社会事务、解决社会矛盾。加强政府法制工作，提高立法质量，强化行政执法监督和行政复议。自觉接受人大及其常委会的法律监督和政协的民主监督，认真执行人大决议，办理好人大代表议案、建议和政协委员提案。完善新闻发布制度，推进政务公开，提高政府工作的透明度和公众参与度。全面实施“五五”普法规划。

　履行职责，建设责任政府。理顺行政管理关系，深化行政管理体制改革。落实服务承诺制、行政问责制和责任追究制，逐步形成工作目标明确、权利义务清晰、责任追究到位的行政行为责任体系，提高政府执行力。认真解决土地征用、房屋拆迁、拖欠农民工工资等群众反映强烈的问题。严格落实公共安全责任制，建立健全政府应急管理体系。完善公共财政体系，加强财政资金管理，逐步加大公共财政投入，保障公共产品的生产和供给。

　转变作风，建设务实政府。坚持从实际出发，从人民群众利益出发，大兴调查研究之风，实事求是、求真务实，讲实话、重实干、办实事、求实效。精简文件，精减会议，切实改进文风会风。厉行节约，反对浪费，建设节约型机关。认真贯彻实施《公务员法》，进一步完善公务员管理制度，切实加强公务员队伍建设。

　从严治政，建设廉洁政府。完善教育、制度、监督并重的惩治和预防腐败体系，加强党员干部党性锻炼和思想道德修养，筑牢拒腐防变的思想道德防线。全面落实党风廉政建设责任制，建立健全有利于群众支持参与的反腐败工作机制。加强行政监察和审计监督，规范权力运行方式，重点加强对财政性资金、工程项目招投标、经营性土地使用权出让、产权交易、政府采购、重大决策和领导干部经济责任等关系公共利益事项的监督。严厉查处大案要案，严肃查处损害群众利益的不正之风和腐败行为，大力治理商业贿赂，最大限度地减少和防止因干部腐败和作风不正引发的社会矛盾。

　各位代表！我市经济社会发展已进入新的历史时期。我们肩负着光荣而艰巨的任务，肩负着人民的重托。让我们高举邓小平理论和“三个代表”重要思想伟大旗帜，全面落实科学发展观，团结和依靠全市人民，立足新起点，把握新机遇，开拓创新，锐意进取，为推动汕头经济社会又好又快发展而努力奋斗！
